# Evaluation of EEG Signals by Spectral Peak Methods and Statistical Correlation for Mental State Discrimination Induced by Arithmetic Tasks

**DOI:** 10.3390/s24113316

**Published:** 2024-05-22

**Authors:** Daniela Andreea Coman, Silviu Ionita, Ioan Lita

**Affiliations:** 1 Department of Electronics, Computers and Electrical Engineering, National University of Science and Technology POLITEHNICA Bucharest, 110040 Pitesti, Romania; ioan.lita@upb.ro; 2 Regional Research and Development Center for Innovative Materials, Processes, and Products for the Automotive Industry (CRC&D-Auto), 110440 Pitesti, Romania

**Keywords:** EEG signal processing, statistical correlation, power spectral density, mental states discrimination

## Abstract

Bringing out brain activity through the interpretation of EEG signals is a challenging problem that involves combined methods of signal analysis. The issue of classifying mental states induced by arithmetic tasks can be solved through various classification methods, using diverse characteristic parameters of EEG signals in the time, frequency, and statistical domains. This paper explores the results of an experiment that aimed to highlight arithmetic mental tasks contained in the PhysioNet database, performed on a group of 36 subjects. The majority of publications on this topic deal with machine learning (ML)-based classification methods with supervised learning support vector machine (SVM) algorithms, K-Nearest Neighbor (KNN), Linear Discriminant Analysis (LDA), and Decision Trees (DTs). Also, there are frequent approaches based on the analysis of EEG data as time series and their classification with Recurrent Neural Networks (RNNs), as well as with improved algorithms such as Long Short-Term Memory (LSTM), Bidirectional Long Short-Term Memory (BLSTM), and Gated Recurrent Units (GRUs). In the present work, we evaluate the classification method based on the comparison of domain limits for two specific characteristics of EEG signals: the statistical correlation of pairs of signals and the size of the spectral peak detected in theta, alpha, and beta bands. This study provides some interpretations regarding the electrical activity of the brain, consolidating and complementing the results of similar research. The classification method used is simple and easy to apply and interpret. The analysis of EEG data showed that the theta and beta frequency bands were the only discriminators between the relaxation and arithmetic calculation states. Notably, the F7 signal, which used the spectral peak criterion, achieved the best classification accuracy (100%) in both theta and beta bands for the subjects with the best results in performing calculations. Also, our study found the Fz signal to be a good sensor in the theta band for mental task discrimination for all subjects in the group with 90% accuracy.

## 1. Introduction

The arithmetic calculation task elicits special interest in the field of neuroscience and cognitive psychology. Neuroimage research shows that arithmetic tasks involve several processes and activate more neural areas of the brain. Thus, mathematical tasks involve memory, attention, and algorithmic and logical processing. It is also fundamental to the perception of numbers (the size and cardinality of the number), the manipulation of numerical quantities, and decision-making for issuing the final result [1].

The ability to perform arithmetic calculations, investigated with the EEG technique, provides information about possible cognitive disorders such as dyscalculia (a specific learning disorder), ADHD (a specific attention disorder), depression, and stress. The data obtained from the mental arithmetic task serve as the basis for designing neurofeedback and brain–computer interface (BCI) systems. 

In order to highlight mental activity during cognitive tasks associated with simple mathematical calculations based on EEG, two conditions are necessary: to acquire EEG signals from a representative sample of subjects and to apply appropriate signal analysis and classification methods to discriminate mental states with and without mental load.

Generally, EEG experiments are laborious because they involve a relatively large number of subjects, a consistent volume of signals acquired at various points of the head, and the accuracy of test conditions. On the other hand, the group of test subjects must be selected according to various criteria (gender, age, level of education, and possibly others).

Therefore, we proposed to analyze EEG signals within the experiment included in the PhysioNet database by (i) using statistical correlation between all pairs of acquisition signals and (ii) determining the size of the spectral peak in three specific bands (theta, alpha, and beta) for all acquisition EEG signals. The analysis of the results was conducted comparatively from the group with subjects carrying out a mental task (named T (for “task”)) and from the same group without a task, named R (for “resting state”).

The primary goal of this study was to conduct a systematic analysis of EEG signals to discriminate between the two mental states mentioned (R vs. T). We aimed to validate our findings by corroborating them with the existing literature on brain neurodynamics in the functional areas responsible for number manipulation and arithmetic operations. First, our study develops classification algorithms, defining an original method for selecting correlation thresholds and spectral peak limits for mental state discrimination and classification. Defining optimal thresholds and addressing potential intersubject variability is the priority for our secondary objective. This paper thus provides a more nuanced understanding of changes in brain activity during mental tasks compared to previously mentioned approaches.

The idea is not only to determine “where” (topographically) the differentiation of mental states occurs at the surface of the cerebral cortex, but also “why” (neuronal connections) this change takes place. This goes beyond simply classifying brain states (relaxed vs. stressed).

The analysis tools we used are simple and easy to apply, requiring neither substantial computational effort nor the use of ML techniques that are more complex and challenging to interpret. The sample size (up to 40 subjects) also fails to warrant the use of ML techniques, which require large volumes of data for training and validation.

In addition, the choice of the correlation threshold for the Pearson coefficient, as well as the acceptable level of spectral variation interval overlap, allows the analyst to control the accuracy of the interval limit comparison method. The analyst can directly establish the accepted level of confidence.

Complex machine learning models are efficient for classification tasks, but are totally non-transparent in the internal training mechanisms. Thus, they do not offer an advantage for understanding the underlying neural mechanisms related to the mental states of our study. We therefore chose a balance between three basic criteria—ease of use, interpretability, and sufficient accuracy—to confirm our results.

### Contributions

We summarize the main contributions of this work as follows:

- Original algorithms were implemented to establish methods for selecting correlation thresholds and spectral peak limits for mental state discrimination.
- The correlation between signals in the theta and beta bands of the most likely combinations that are useful in discrimination was determined in the presence and absence of mental load.
- Through the tests, we established that the relevant correlation threshold is 0.5.
- The spectral peak criterion in the theta and beta bands was used to classify mental states using the baseline method of boundaries.
- The results obtained are compared with findings from the existing literature.

The rest of this paper is structured as follows: Section 2 provides an overview of the papers strongly related to brain behavior in arithmetic tasks. Section 3 describes the principle of the method applied for the experimental data, the arguments based on brain neurodynamics, and the processing methods and algorithms used for the classification of mental states. In Section 4, the data obtained through the proposed algorithms are analyzed in order to identify relevant EEG signals in the discrimination of mental states. In Section 5, the results obtained are analyzed, correlating the brain areas that are activated in mental tasks with the identified EEG signals. Section 6 contains this paper’s conclusions.

## 2. Related Works 

A significant proportion of recent papers approach the electrical activity of the brain in the presence of mental stress induced by mathematical tasks. EEG analysis in these studies involves methods in the time and frequency domains or combined spectral and statistical methods, using machine learning (ML) techniques. In the following, we present, in summary, approaches from papers in the mainstream of recent publications on this topic.

In [2], the EEG data are filtered in five standard sub-bands of the brain waves extracting features such as energy, entropy, average, and Euclidean norm. The aim is to discriminate the resting state vs. the state induced by arithmetical cognitive tasks with a support vector machine (SVM), Decision Tree (DT), and Quadratic Discriminant classifiers (QDs).

In [3], the EEG signal is decomposed into its frequency components using the Fourier Decomposition Method, and the energy, entropy, and variance of the signals are calculated for each extracted component. The authors employ the Kruskal–Wallis method to select features that are statistically different between mental arithmetic tasks and the relaxed state. The SVM classification method with a cubic kernel yielded the best classification results distinguishing mental states.

In [4], discriminating stress levels in arithmetic computation tasks is proposed. The authors used a combination of frequency domain features based on frequency spectral distribution and time domain features (root mean square amplitude (RMS) and an autoregressive model (AR)). Five ML classifiers (LDA, KNN, Random Forest, cubic and linear SVM) were used to detect stress and non-stress states as well as to distinguish between stress levels with a minimum number of electrodes. Thus, two EEG channels with the best performance were identified for detecting stress and non-stress in the prefrontal area, and three EEG channels were identified for stress levels in the prefrontal and frontal areas.

In [5], the authors analyze EEG signals by windowing them in frames of 500ms using the time–frequency distribution (TFD), evaluating the instantaneous frequency (rate of change of signal frequency over time) in each time window, and extracting the mean and standard deviation statistical features for the data of each frame. They proposed a generalized structure of the LSTM architecture to avoid over-fitting, including dropout layers, aiming at classifying mental tasks. An intensification of activity in the frontal, temporal, and occipital lobes was observed during the performance of mental arithmetic tasks.

In [6], the characterization of EEG time series is conducted in terms of variability, irregularity, and signal complexity using the following statistical measures: Sample Entropy, Approximation Entropy, Dispersion Entropy, Permutation Entropy, and Slope Entropy. Between the mentioned entropy types, the Slope Entropy obtained higher classification accuracy for all RNN models (LSTM, BLSTM, and GRU) used. It was found that EEG signals in the median and right visual areas are significant for the classification of tasks during the mental arithmetic process. Similarly, the Long Entropy feature was evaluated for eight different types of ML classification in [7]. The best performance was obtained by using the ensemble classifier. The study was based only on the performance of the classifiers, while no discriminating regions or electrodes were mentioned.

In [8], statistical characteristics (medians) were extracted from PSD (power spectral density), which reflects the intensity of neural oscillations and their coherence to indicate the level of synchronization in different regions. Additionally, DFA (Detrended Fluctuation Analysis) scaling exponents were utilized, which expressed the stability of fluctuations in neurons in the temporal domain. The results were evaluated comparatively for the three types of characteristics applied both in the resting state and during mental workload, to identify the neural processes that lead to the performance of the task. In the theta and beta bands, changes are evident depending on the two states in different brain regions.

Papers [2,3,4,5,6,7,8] all used the same PhysioNet public databases.

Generally, these studies provide a comparative overview of classification methods, identifying specific EEG signals suitable for mental state classification.

Below, a number of studies are presented that featured their own experiments, particularly having the arithmetic task as a type of stressor.

Most research focused on utilizing frequency domain analysis to discriminate between brain states associated with arithmetic tasks, while aiming to minimize the number of electrodes required. Power spectral density (PSD) has the highest usage rate in feature extraction, followed by different types of entropy [9].

In [10], a single frontal electrode (Fp1) mental arithmetic task was investigated. The PSD was estimated with the Welch method, obtaining 65% and 75% accuracy, using a support vector machine (SVM) classifier.

The attempt to minimize the number of electrodes sometimes led to a decrease in system performance, as in [4]. For example, in [11], for a total number of 14 EEG recordings, spectral features were extracted with the SFFT (Short Fourier Transform) method for an n-back test and mental arithmetic task, with an SVM classifier. An 80% accuracy was obtained using the T8, T7, O1, and F7 channels, and an 89% accuracy resulted when all 14 electrodes were used.

The combination of statistical characteristics, PSD, coefficients of the autoregressive model (AR), and the generalized Higuchi fractal dimension spectrum (GHFDS) were used in [12]. Through GHDS, the unpredictable action of brain activity is estimated. An 84.15% accuracy was obtained in discriminating states of relaxation vs. arithmetic task for single-channel F8 and 97.87% for F8, F3, O2, and AF3.

The analysis that focused on the prefrontal region in [13] used a functional connectivity method by Magnitude Square Coherence (MSC), which reflected the degree of synchronization of electrical activity between signals. A decrease in functional connectivity in the alpha band was observed in the context of the effects of arithmetic stress. Also, in the frontal and prefrontal regions, the most intense discriminatory connections were observed between the resting state and the state involved in the arithmetic task in the beta2 band (15–22 Hz) [14]. This study also evaluated connectivity with a causal relationship between different brain areas through Generalized Partial Directed Coherence (GPDC), with an accuracy of 89%, as well as through an SVM classifier.

The above-mentioned papers analyze the performance of the selected methods in the classification of mental states and explore the brain regions that are most relevant to the activity induced by the task. The main focus is on classifying brain states (resting vs. arithmetic task) using ML, neglecting a more comprehensive analysis of the underlying neural processes associated with task difficulties. These studies also prioritize achieving high classification accuracy in discriminating states, mostly neglecting more nuanced changes in brain activity.

Choosing the right method for feature extraction is always a challenge in the EEG field [15,16]. Most of the research mentioned uses methods of extracting features from the EEG signal that require a large amount of calculation, such as entropies used in [6,7]. The fractal dimension from [12] also contributes to this computational load, limiting its applicability in real time. The AR method minimizes spectral losses, but model instability problems may occur if the AR order is selected incorrectly. PSD meets challenges in choosing the size of the analysis window. Statistical methods have increased speed in extracting features, but they are sensitive to data that presents artifacts, which would result in large variations in the data.

In a recent work [17], it is pointed out that the results of the studies in this area are not uniform, and therefore, further processing and interpretation are needed to consolidate and complete the existing results.

## 3. Principle of the Method

### 3.1. The Structure of the Experimental Data

Although there has been increased interest in studying arithmetic processing in the brain in recent years, there are still few databases with acquired signals for such experiments. In Table 1, two databases are provided that contain experimental data for mental activities associated with arithmetic tasks.

In this paper, the experiment from the PhysioNet [18] database was selected for analysis. We decided this because the PhysioNet database provides more measurement channels (19 vs. 6), a larger number of subjects (36 vs. 7), and a doubled sampling rate compared to the Keirn and Aunon database [19].

The experiment from PhysioNet was conducted in laboratory conditions on an initial number of 66 subjects from which, in the end, a representative working group of 36 subjects was retained.

The inclusion criteria were normal visual acuity and the absence of cognitive impairments. The exclusion criterion was dependence on psychotropic drugs [20]. The EEG recordings were processed to remove power line noise by applying a notch filter (50 Hz) and to remove physiological artifacts (eye movements, muscle, heart activity) using the ICA (Independent Component Analysis) technique. Table 2 shows the conditions and particularities of this experiment.

The EEG recordings for the 19 measurement channels have two specific lengths dictated by the duration of the recording in the two situations: in the absence of tasks (R), the acquisition time was 180 s; in the presence of the mental task (T), the recording time was 60 s.

Under these conditions, the signals from the first category contain 180 × 500 samples/s = 90,000 samples each, and the signals from the second category have 60 × 500 samples/s = 30,000 samples each.

The subjects perform an arithmetic task consisting of serial subtraction operations between four-digit numbers and two-digit numbers, for example, “3141 − 42 = 3099” and then the second subtraction, “3099 − 42=3057”, and so on.

The experiment aimed to emphasize another characteristic of the process by quantifying the accuracy of performing operations. Thus, depending on the performance obtained regarding the number of correct operations performed in the allotted time (60 s), the 36 volunteer subjects were separated into group “G” (for “Good”), who easily performed more correct operations, and group “B” (for “Bad”), who correctly performed a smaller number of operations. Subject 31 (group G) and Subject 4 (group B) were excluded because the duration of their signal recordings was shorter.

The hierarchy of subjects based on their performance is shown in Table 3. Beyond grouping into G and B, we separated a particular group from G, which we designated as G*, which included subjects with a performance score above the group average equal to 22.08 and a standard deviation less than 3.00. Thus, group G* includes the first 11 subjects whose performance score is between 34.59 and 26 (see the shaded rows in Table 3).

### 3.2. A Few Aspects of Brain Neurodynamics

**Figure 1:**
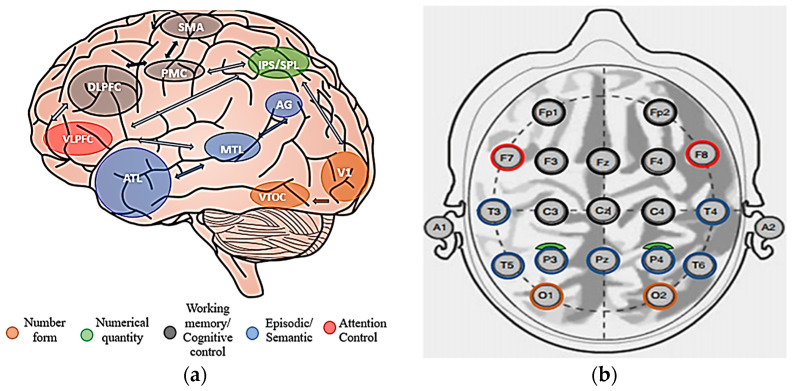
Correspondence of brain areas of interest with EEG measurement points. (**a**) Schematic circuit of neurocognitive nodes in arithmetic process, adapted from [1]. (**b**) Electrode placement in 10–20 system, colored according to the cognitive nodes involved in the arithmetic process, adapted from [25].

The interpretation of EEG signals in the context of mental arithmetic tasks must be corroborated with information about the neurodynamics of the brain in such states. The neurodynamic processes specific to the mental stress induced by the arithmetical tasks are located in different areas of the brain, which helps to choose the topology of the measurement points. Reference [1] gives us a general picture of the areas of the cortex involved in the mental processes in connection with computation, as shown in Figure 1a.

Studies based on functional magnetic resonance imaging (fMRI) have identified cortical and subcortical regions, called nodes [21], with specific functions in the involvement of arithmetic tasks. These regions, through neural processes, direct certain electrical circuits depending on the type of arithmetic operation (+, −, ×, /), the complexity of the problem by manipulating the number of operands [22], and the differential processing of some correct and incorrect equations [23].

Regarding the topology of the measurement points, evidence of the brain regions most involved in the arithmetic task was sought. Neuroimaging studies have located the anatomical functional regions that include and largely overlap with Brodmann’s areas, thus making it possible to identify the closest EEG electrodes in the 10–20 system.

These electrodes are, in most cases, involved in studies on the mental arithmetic task [24]. This is highlighted in Figure 1a, with a representation of the EEG points of the 10–20 system in Figure 1b.

Electrodes Fp1, Fp2, F3, F4, and Fz correspond to the DLPFC area (dorso-lateral prefrontal cortex), and Brodmann areas 9 and 46, respectively [26]. DLPFC represents the area most frequently related to executive functions such as working memory (temporary storage and manipulation of information), selective attention (filters, distractions, letting important information pass [27]), and cognitive flexibility, i.e., the ability to switch between different tasks or mental strategies. Additionally, inhibition, i.e., the suppression of unwanted thoughts, actions, or emotions, alongside planning and making decisions based on available information are also associated with DLFPC.

An experiment presented in [28] using the tDCS (Transcranial Direct Current Stimulation) technique focused on a decision-making task under conditions of risk and ambiguity. It was shown that F3 and F4 mainly captured the activity in the DLFPC area when risky situations occurred. Fp1 and Fp2 received information from the neighboring prefrontal area that corresponds to the OFC area (orbito-frontal cortex) sensing the ambiguity in the process of real-time decision-making.

The VLPFC area (Ventrolateral Prefrontal Cortex) has a crucial function in clarifying knowledge (disambiguation) in situations where initial processing generates multiple interpretations of information [19]. EEG channels F7 and F8 are influenced by the activity in the VLPFC area [13], as well as Brodman areas 47, 45, and 44 [29]. Studies also suggest that the right and left sides of the VLPFC have distinct functions.

EEG channels C3, C4, and Cz capture signals from the SMA (Supplementary Motor Area) and PMC (Primary Motor Cortex) area close to Brodman areas 6 and 4 [30,31], with a role in planning cognitive actions as well as the intention of speech.

Channels T3, T4, T5, and T6 capture the activity from the subcortical areas MLT (medial temporal lobe) and ATL (Anterior Temporal Lobe). The MLT is a subcortical region in the hippocampus and parahippocampus, Brodmann areas 34 and 28 [32], which are reflected in the temporal areas. MLT is also called the “hub of hubs” in the literature, being associated with a series of cognitive functions such as memory, episodic recall, spatial information storage, and learning [33]. It also receives visual and auditory information which it then integrates and transmits to other regions.

The ATL area, which corresponds to Brodmann area 38, together with the anterior parts of Brodman areas 20, 21, and 22, is considered a semantic processing unit according to [34].

Channels P3, P4, and Pz are projected on the AG region (Angular Gyrus), which is part of the parietal lobe (Brodman area 39). It is located at the connection between the temporal and occipital lobes, being a cortical node involved in multiple functions: episodic memory, the retrieval of facts in calculation processes, the retrieval of verbally expressed numerical facts, visual–spatial processing, and abstract concepts [22,35,36,37]. The area is more active in the left hemisphere [37], effectively in the central–parietal area on the CP5 channel [38], with, in our case, the closest electrode being P3.

In the proximity of the AG region, there are two cortical portions—the IPS (intraparietal sulcus) and SPL (superior parietal lobe)—in the parietal area, more precisely in the dorsal lateral parietal cortex. This node is involved in numerical recognition and even in evaluating the difficulty of a problem [39]. Together with visual areas, they help to create visual and spatial representations of numerical quantities [1].

In the occipital region, the primary visual area (V1, Brodmann area 17), and the ventral temporal–occipital cortex (VTOC, Brodmann area 37) [40] are responsible for processing numbers and generally evaluating numerically expressed quantities [41].

### 3.3. Processing Methods Used

#### 3.3.1. Statistical Correlation

The types of correlations used in EEG analysis reported in the literature are Pearson correlation, Spearman rank correlation, and Kendal rank correlation. Between these, Pearson correlation is more suitable for the analysis of EEG data [42], being used as a method to investigate functional connectivity, as an expression of linear interactions between different brain regions [43].

Experimental research indicates that for EEG signals, the Pearson correlation coefficient can reach maximum values around 0.8 [44]. However, this value cannot serve as a reliable discriminator for mental states due to its variability across subjects.

The correlation between signals is an indicator that generally strengthens the decision-making capacity of a system in the sense of highlighting reliable triggers for certain mental states. 

In [45], the testing of the functional connectivity of working memory in the theta, alpha, beta, and gamma bands is presented. This was achieved by retaining visually presented information related to a control state with brain activity during a resting state. Inter-hemispheric connectivity was found in the temporal and occipital lobes (T5/T6 and O1/O2) in the beta band within the context of working memory.

In another study, the Pearson correlation coefficient is utilized to evaluate mental fatigue in the theta, alpha, and beta bands.

This evaluation involves arithmetic calculations (+, −, ×, /) conducted in experiments lasting a total of 3 min. These experiments include equations with an allocated duration of 8 s each, as reported in [46]. In this case, the Pearson coefficient revealed increases in the theta and beta bands compared to alpha (through the *p*-value from the *t*-test) during the arithmetic task.

The Pearson correlation coefficient was also used in [47] for the study of the EEG signals involved in a visual stimulation experiment.

For two discrete signals, as time series ${{(s}_{1})}_{k}$ and ${{(s}_{2})}_{k}$, for *k* = 1, …, n, the Pearson correlation coefficient is defined by the following equation:

(1) $$R_{s_{1},s_{2}}=\frac{\sum_{k=1}^{n}({\left(s_{1}\right)}_{k}-\bar{s_{1}})({\left(s_{2}\right)}_{k}-\bar{s_{2}})}{n\sigma_{s_{1}}\sigma_{s_{2}}}$$

where

$\bar{s_{1}}$, $\bar{s_{2}}$ are the average values of the signals;

$\sigma_{s_{1}},\sigma_{s_{2}}$ are the standard deviation of the two signals;

n is the number of considered samples.

In our experiment, we used the Pearson correlation coefficient to investigate the statistical correlation between all the pairs (combinations) of the 19 raw EEG signals filtered in each of the three bands, theta, alpha, and beta. The study was organized for the following cases:

- The correlation of the 19 raw signals without the task (R) in all their possible combinations, taken two by two, that is, 171 ($C_{19}^{2}$);
- The correlation of the 19 raw signals with the task (T) in all their possible combinations, taken two by two, that is, 171 ($C_{19}^{2}$);
- The correlation of the 19 filtered signals in the theta, alpha, and beta bands without the task (R) in all their possible combinations, taken two by two; this means 171 ($C_{19}^{2}$) × 3 = 513 data;
- The correlation of the 19 filtered signals in the theta, alpha, and beta bands with the task (T) in all their possible combinations, taken two by two, resulting in a total of 171 ($C_{19}^{2}$) × 3 = 513 data.

#### 3.3.2. Spectral Method

The investigation tool used is the Fourier Transform (FT) applied to EEG signals to estimate their spectral power in the frequency domain. We use this technique to extract a measure of the dominant level of spectral power in three frequency bands dedicated to the study of brain waves: theta (4–8 Hz), alpha (8–13 Hz), and beta (13–30 Hz).

**Figure 2:**
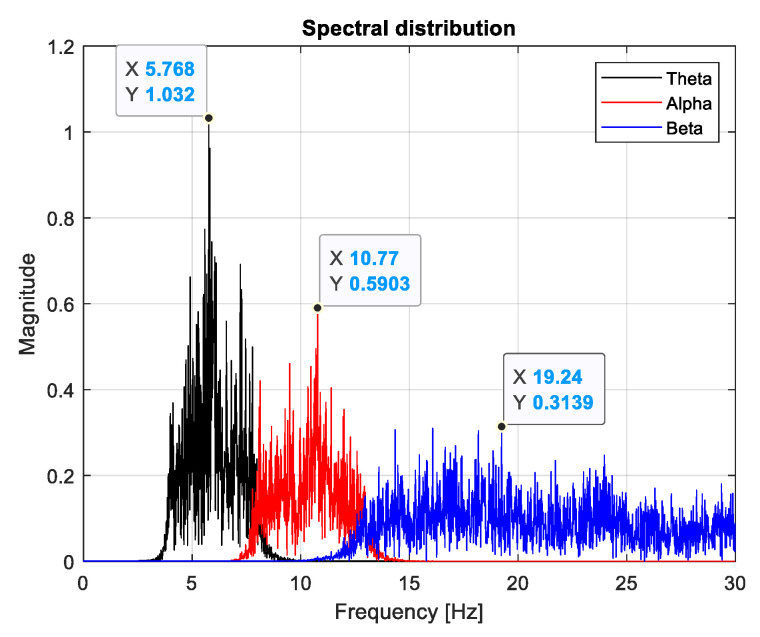
Spectral density in three bands and recorded spectral peaks.

In our study, we used an algorithm for filtering in the three bands and extracting the value of the spectral peak with the identification of its corresponding frequency in each band. Under these circumstances, IIR digital band-pass filters of order 20 were designed. Figure 2 shows a representation of the spectrum along the three bands for the F7 signal measured during task performance for subject number 27, who has the maximum performance score.

The algorithm was applied for the following cases:

- For the whole batch of subjects without the mental task (R);
- For the whole batch of subjects with the mental task (T).

Thus, for each case, 34 subjects × 19 channels × 3 bands = 1938 data will be determined, for both cases (R and T), leading to 3876 data.

The data analysis operations were carried out automatically with a Matlab R2020a application program. The data of interest were saved in a database file.

#### 3.3.3. Discrimination of Mental States

**Figure 3:**
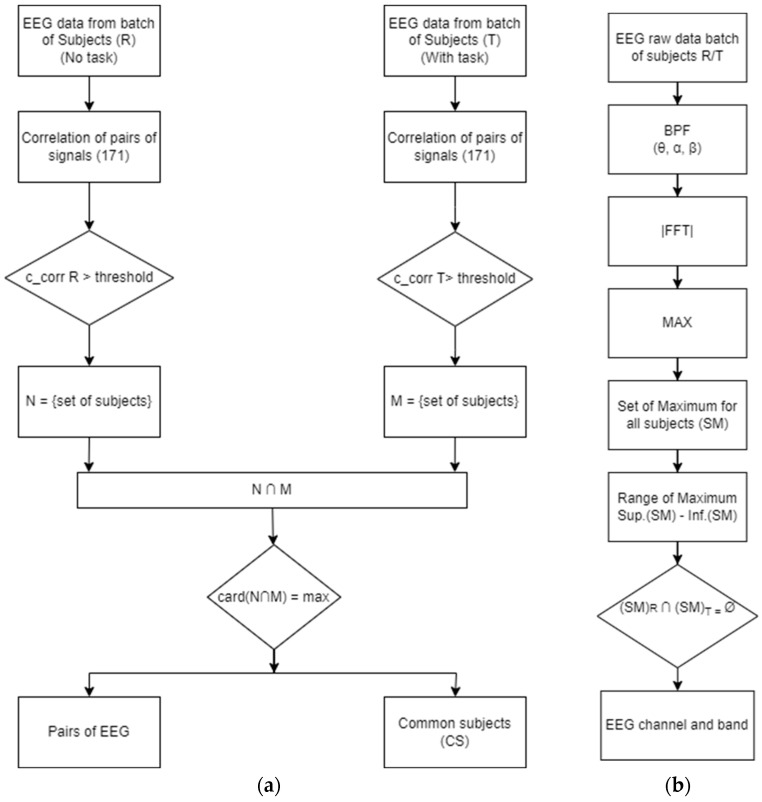
Algorithms implemented for state discrimination: (**a**) scheme of the evaluation principle based on correlation; (**b**) scheme with the principle of evaluation by spectral maxima.

The detection of mental states based on the statistical correlation coefficient is carried out with the algorithm in Figure 3a. It provides two pieces of information: (i) the pairs of EEG signals that meet the conditions for a certain correlation threshold, and (ii) the number of subjects that fall into this selection.

The principle of the algorithm is implemented as follows: the database of EEG signals is read, the Pearson correlation is calculated for all pairs of signals, and a corresponding condition is tested for a certain correlation threshold. Therefore, a lot of subjects that meet the criterion are obtained. This results in the set N of subjects confirming the state of relaxation (R) and the set M of subjects confirming the state with mental load (T) by fulfilling the system of complementary conditions, as follows:

(2) $$\left\{\begin{array}{c} c_{corrR}>threshold\\ c_{corrT}<threshold \end{array}\right.$$

The discriminator described by Equation (2) is built on the following assumption: On the one hand, in the relaxed state (R), functional connectivity in the brain is weaker and the number of pairs of correlated signals above a certain threshold might be higher. On the other hand, in the mental load state (T), functional connectivity increases and correlations might fall below a certain threshold.

Ideally, the two sets should coincide, which would confer a degree of confidence of the discriminator of 100%. Practically, we expect state classification errors in the following sense:

- Not all subjects who do not respect the first inequality will fulfill the second one;
- Not all subjects who do not respect the second inequality will fulfill the first one.

So, classification error is when a subject in state R is classified in state T and vice versa. We will consider the classification of a subject in state T as being in state R as a false positive classification error.

Thus, in practice, we put a condition so that card (N ∩ M) is as large as possible, which would minimize the classification error, including for false positive situations. This condition effectively maximizes the group for which condition (2) is fulfilled. Finally, the algorithm provides the common subjects whose states are correctly classified and the pairs of correlated channels to which discrimination occurs for the correct classification.

The classification algorithm based on the size of the spectrum peak is illustrated in Figure 3b. It includes filtering all signals in the theta, alpha, and beta bands and applying the Fourier Transform for each band followed by recording the spectral power maxima. This sequence of steps applies to the group of subjects in the situations without mental load (R) and with mental load (T).

This results in two sets of spectral maxima—for the group without the load (SM)_R_ and for the group with the load (SM)_T_. The range of maxima is determined by evaluating the superior and inferior of each set of spectral maxima. The discrimination is effectively achieved by the method of comparing the limits of the two sets of the maxima (for state R and state T) for each EEG signal. For a perfect classification, the two domains must be disjointed; that is, the intersection of the sets of maxima must be the set Ø. This is expected to happen for certain EEG channels in certain bands of signals.

## 4. Data Analysis

Applying the algorithms presented in the previous section generated a considerable volume of data to analyze. In this section, the data obtained will be summarized and systematized in such a way that the comparison between the tests with a mental task (T) and those without a task (R) will be as facile and relevant as possible.

### 4.1. The Correlation Study

The conjugate selection condition using the correlation coefficient described by Equation (2) will be customized for a certain correlation threshold value. In practice, the average value of the correlation coefficient is considered to be in the range of 0.5–0.7, as is reported in [40]. We run the proposed algorithm for the threshold value equal to 0.5, which leads to the following conditions:

(3) $$\left\{\begin{array}{c} c_{corrR}>0.5\\ c_{corrT}<0.5 \end{array}\right.$$

The algorithm presented in Section 3.3.3 was separately applied for the group of subjects with good scores (G, 25 subjects) and for the group with bad scores (B, 9 subjects), across the three frequency bands.

**Figure 4:**
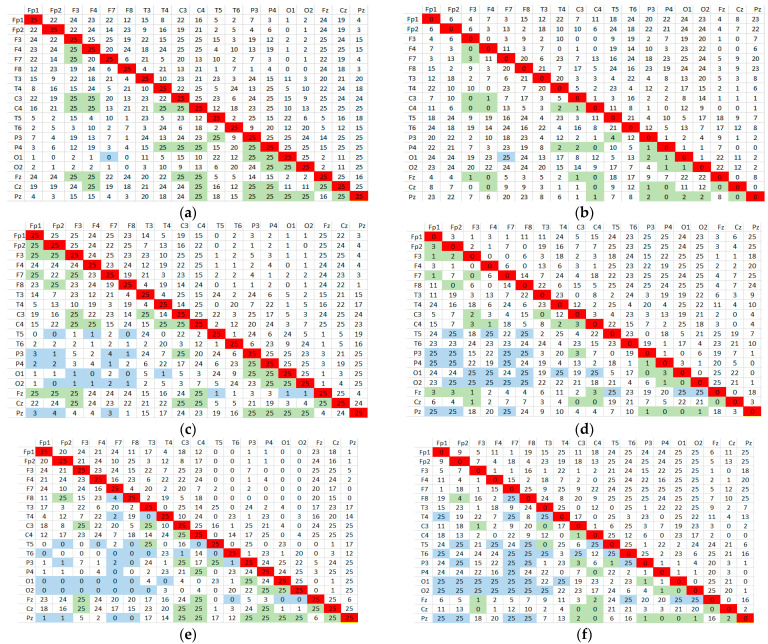
Matrices with the number of subjects fulfilling the correlation threshold condition for group G in (**a**,**c**,**e**) theta, alpha, and beta bands with c_corr R > 0.5 and in (**b**,**d**,**f**) theta, alpha, and beta bands with c_corr T < 0.5, where green and blue mark the extreme values in the matrices.

**Figure 5:**
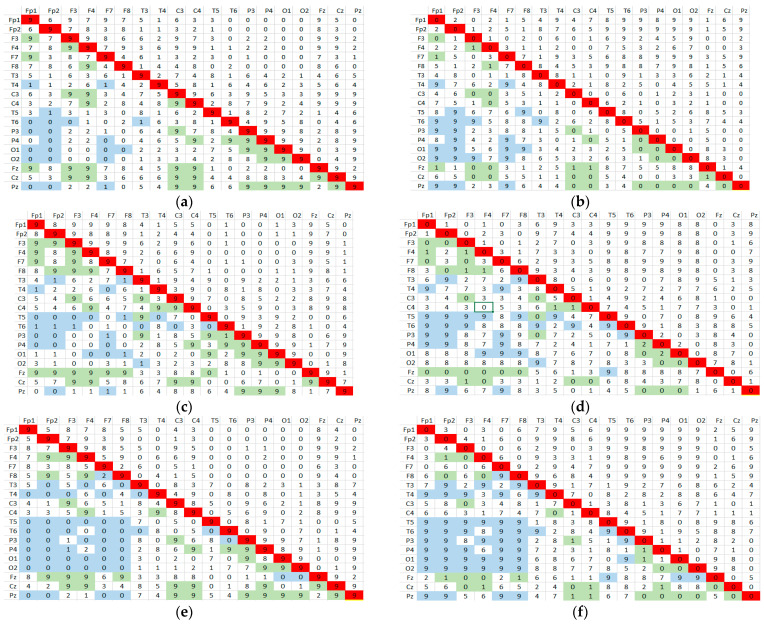
Matrices with the number of subjects fulfilling the correlation threshold condition for group B in (**a**,**c**,**e**) theta, alpha, and beta bands with c_corr R > 0.5; (**b**,**d**,**f**) theta, alpha, and beta bands with c_corr T < 0.5, where green and blue mark the extreme values in the matrices.

The algorithm calculates, successively for each subject, the Pearson correlation coefficient according to Equation (1), between all combinations of the 19 EEG signals, excluding trivial combinations (i.e., a signal correlated with itself). Any combination of signals will be indexed by one unit if threshold condition (3) is met. Using the matrix representation in 19 × 19 combined signals, after evaluating the entire group of subjects, each element of the matrix will represent the number of subjects for whom the hypothesis is verified. These results are shown in Figure 4a–f for group G and Figure 5a–f for group B, representing the matrices containing the number of subjects that meet the correlation threshold condition for all combinations of EEG channels in the three bands.

Let us now explain the significance of these results. In Figure 4, the arrays on the left side (Figure 4a,c,e) denote the number of subjects meeting the correlation criterion for the relaxed state (R) in each pair of correlated signals. For each specific frequency band, the number contained is noted to differ one pair from the next, from 25—the total number of subjects in the group—to 0; that is, no subject met the condition. These extreme values are marked (shaded) in their boxes in green and blue. Now, looking at the matrices on the right side (Figure 4b,d,f), we have the result of the complementary test applied to the subjects in the state with mental load (T). We also see numbers from 25 to 0 representing subjects that meet the conjugate criterion, with their positions in the matrix also marked in green and blue. Comparing pointwise sets of marked values between right and left corresponding matrices, we observe exclusive, complementary, or significant disjunction. This meant that the correlation criterion for those signal pairs could not discriminate against mental state in this experiment.

Therefore, we should pay attention to those pairs of correlated signals for which the number of subjects in the matrix on the right is as close as possible to that in the matrix on the left. These situations represent a compromise that leads to maximizing the number of subjects who simultaneously meet condition (3), i.e., the one that provides MAX(card(N ∩ M)).

Table 4 summarizes the number of signal pairs for each frequency band for which the correlation threshold condition is met in both subject groups, G and B. These were obtained by counting the marked boxes in the matrices. An analysis of the sensitivity of the method in R states was also included, which are above the threshold of 0.5 to 0.8 (the last value above which no combination of signals is obtained).

The correlation drops dramatically in the R (resting) state after the threshold of 0.5. Only in the alpha band, specific to the relaxation state, are there pairs of correlated signals with a high threshold—namely, 0.8. This suggests that the brain might be more coordinated in its activity during a relaxed state. 

It is observed that most of the total signal combinations ($C_{19}^{2}$ = 171) do not correlate for the threshold imposed for all 25 subjects from group G and the 9 subjects from group B. Moreover, it was found that the fulfillment of the correlation conditions decreases as the threshold value is higher.

Next, we present a particular analysis of correlation using data from correlation matrices in the beta band. A perfect discrimination would be when certain pairs of signals fulfill both conditions for all subjects in the group. In other words, for the confidence level of the criterion of 100%, the above conditions should be fulfilled simultaneously by all 25 subjects in group G and the 9 subjects in group B. Thus, comparing the numbers from the positions highlighted in the matrices on the left and on the right in Figure 4 and Figure 5, we rather observed a complementarity, but not a numerical concordance. The differences between the number of subjects who meet the condition c_corr R > 0.5 and those who meet the condition c_corr T < 0.5 is quite large for both groups G and B.

**Figure 6:**
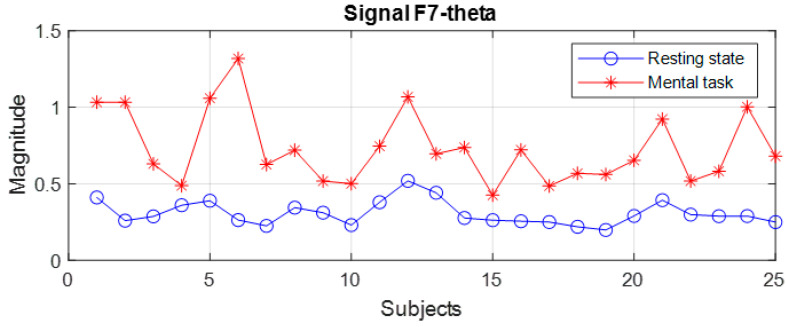
Spectral peaks for the F7 signal in the theta band of subjects from group G.

For example, in Figure 4, for the theta band for the F7–F3 signal pair, all 25 subjects meet the condition for the no-task condition (c_corr R > 0.5), but only 3 subjects meet the condition for the task condition (c_corr T < 0.5). The difference is even more drastic in the alpha band: 25 to 0, respectively, in beta band 24 to 1. The situation is similar for group B in Figure 6.

However, we notice that, for some combinations of signals, both correlation conditions are met by numerically balanced groups and subjects, which could highlight a higher degree of confidence in the discrimination of states. Such combinations are identified and summarized in Table 5. It can be seen that in the correlation matrices in the beta band, four pairs of signals were found that maximize the condition for card(N *∩* M) in accordance with the algorithm presented in Figure 3a.

For the first two signal combinations in Table 5, the subjects identified in each set are nominated in Table 6.

As can be seen in Table 6, for the experimental group considered “Good”, composed of 25 subjects who answered with good scores, the confidence percentage of the discriminator of the mental task through correlation depends on the number of common subjects (CSs) reported versus the total number of subjects in this group. As the matter of fact, the obtained data show that, for the pair of signals F7–F4, this is only 6/25 × 100 = 24%; for the pair of signals F8–F3, it is 7/25 × 100 = 28%.

In conclusion, we note that the correlation of signals as a discriminator of states is not reliable.

However, if we wanted to follow a possible connection between the subjects from the common group (CS) and the performance score obtained in the test by each of them, we see the hierarchy in Table 7. Here, to, is a weak connection between the discriminable subjects and their score in the mental task test. In other words, the subjects in the common group do not all have the best scores, the average score for the whole batch being 22.08. It is observed that only three subjects with an above-average score fulfill the correlation conditions at F7–F4, and five subjects fulfill the correlation conditions at F8–F3.

For the group of subjects in the “Bad” category, which includes nine tested persons who obtained scores lower than or equal to 10 points, the same procedure was used to analyze the correlation matrices. The obtained results are summarized in Table 8 and Table 9. The conclusion is that even in their case, the selection criterion based on the correlation coefficient cannot be used, the best confidence percentage being 3/9 × 100 = 30%.

Regarding a possible link between the subjects in the common group and the performance score obtained in the test by each one, we see the hierarchy in Table 10. In this case, there is no obvious link between the discrimination of the task and the score obtained.

### 4.2. Spectral Study

The spectral analysis in the three frequency bands for each EEG signal performed on the two groups of “Good” and “Bad” subjects generated a considerable amount of data, as evaluated in Section 3.3.2, with a volume of 3876. The purpose of this study is to evaluate the ability of the peak spectral power indicator to discriminate based on the presence or absence of the specific mental state imposed by the arithmetic task for the groups of tested subjects.

**Figure 7:**
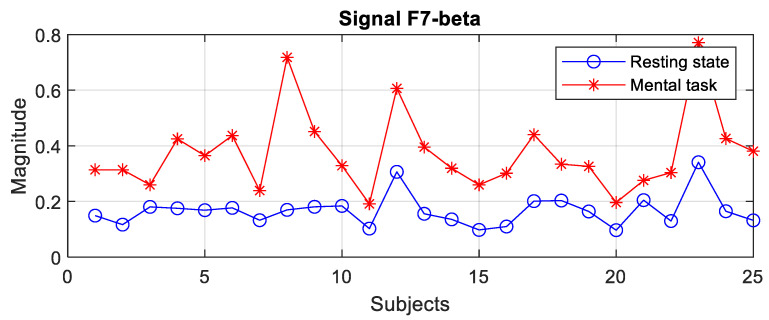
Spectral peaks for the F7 signal in the beta band of subjects from group G.

**Figure 8:**
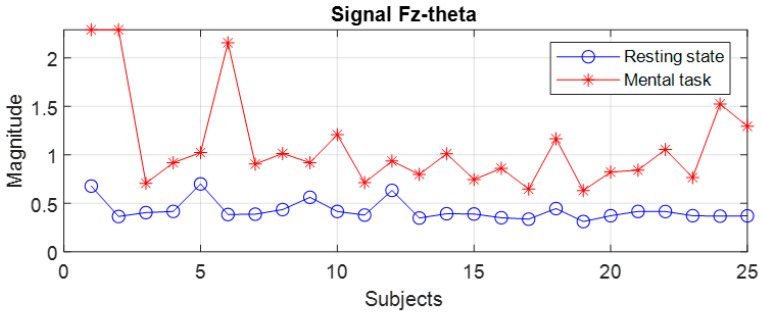
Spectral peaks for the Fz signal in the theta band of subjects from group G.

The file with the spectral data, which was generated with the algorithm presented in Figure 3b, was read with an application program that evaluated the variation domains of the recorded spectral peaks. A first finding is that for the group of subjects with good scores (“Good”), none of the 19 signals allowed the differentiation of states for all 25 subjects, with the variation domains presenting a relative degree of overlap in all frequency bands for a few subjects. The general observation is that the values of the spectral peaks detected in the case of the resting state (R) are lower than in the case of the present mental task (T). However, there are subjects for whom the recorded level of the maximum central power in state R is higher than the smallest spectral peak recorded in state T. For example, even for two signals with the best separation of states, an overlap of the global limits is observed in a few subjects as illustrated in Figure 6, Figure 7 and Figure 8. Subjects 1 to 25 are arranged in descending order based on their performance score obtained in the test. We notice that for the F7 signal in the theta band, there is an overlap in 6 subjects, these being S4, S9, S10, S15, S17, and S22, while in the beta band, the overlap occurs in 14 subjects. For the Fz signal in theta, only two subjects exhibit an overlap. It is observed that the principle of classification based on the boundaries of the ranges of variation in the spectral maxima does not present a 100% degree of confidence.

**Figure 9:**
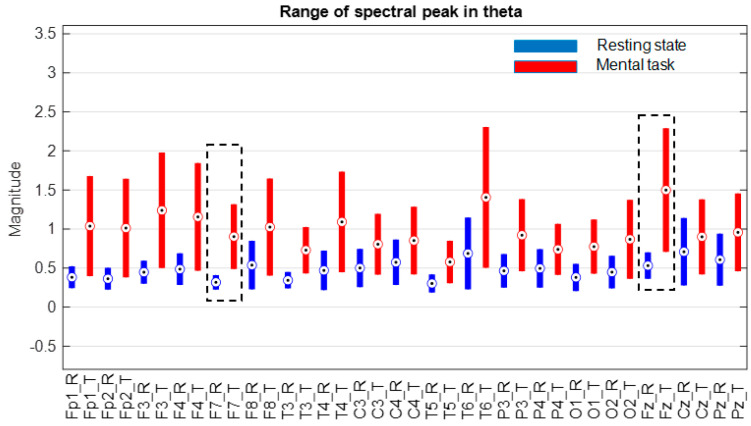
Ranges of variation in the size of the spectral peak theta for subjects in group “G*” with score > 26 on the mental test. Circles indicate the median of the ranges, and the black dashed lines the non-overlapping ranges.

**Figure 10:**
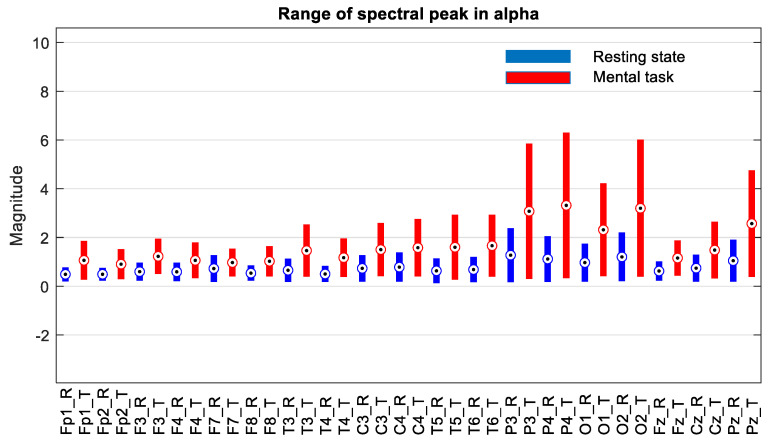
Ranges of variation in the size of the spectral peak alpha band for subjects in group “G*” with scores > 26 on the mental test, where circles indicate the median of the ranges.

**Figure 11:**
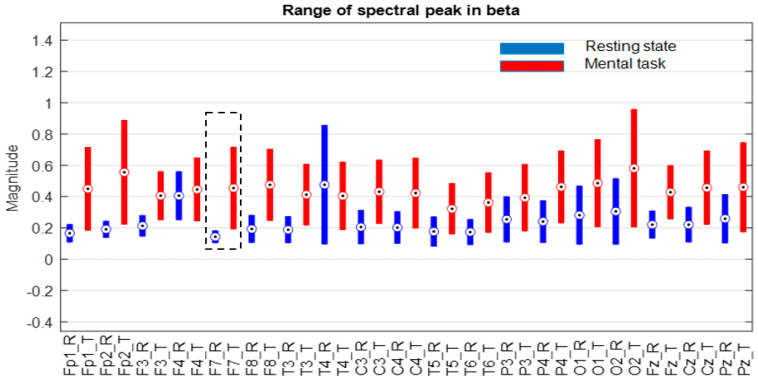
Ranges of variation in the size of the spectral peak in beta band for subjects in group “G*” with scores > 26 on the mental test. Circles indicate the median of the ranges, and the black dashed lines the non-overlapping ranges.

The next step was to consider only one group selected according to the score obtained in the mental test. Thus, we decided to choose only the subjects in group G*, which includes only the first 11 subjects, whose scores fall between 34.59 and 26, as is described in Section 3.1. The evaluation of the ranges of variation in the spectral peaks recorded for these subjects is presented in Figure 9, Figure 10 and Figure 11.

At first glance, we find that, in the theta and beta bands, the ranges of variation and their median values for all signals are significantly higher in the presence of mental tasks. This is the quantitative measure, which alone fails to guarantee the discrimination of mental states. Therefore, we have to check the full disjunction of domains by comparing min–max limits.

The comparative analysis highlights a clear separation of ranges for the F7 signal in the theta and beta bands, as well as the Fz signal only in the theta band, as they are framed by the dashed rectangle in Figure 9 and Figure 11. There are no signals with separability in the alpha band, as can be seen in Figure 10.

Thus, for the F7 and Fz signals in the mentioned frequency bands, it is observed that the separability of the spectral values for the first 11 subjects is 100% fulfilled.

**Figure 12:**
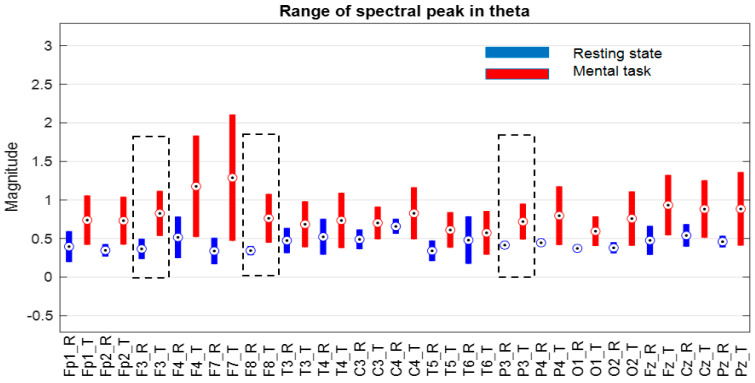
Ranges of variation in the size of the spectral peak in the theta band for subjects of group “B”, where circles indicate the median of the ranges, and the black dashed lines the non-overlapping ranges.

**Figure 13:**
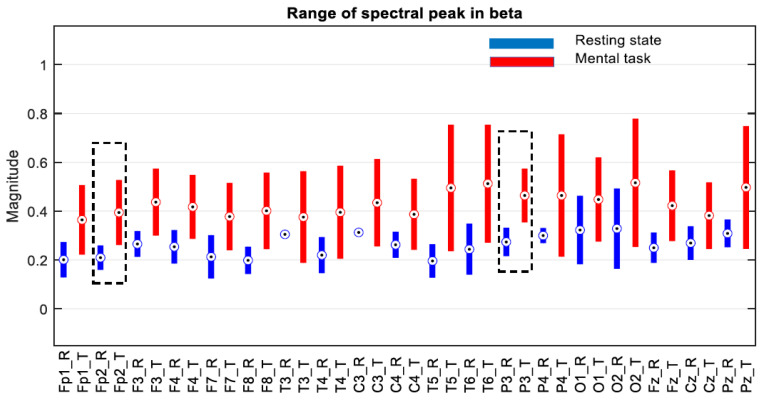
Ranges of variation in the size of the spectral peak in the beta band for subjects of group “B”, where circles indicate the median of the ranges, and the black dashed lines the non-overlapping ranges.

Similarly, group B, which contains nine subjects with low performance scores that were between 10 and 1, was analyzed. The separability of the ranges of the spectral peaks is performed for the whole group based on signals F3, F8, and P3 in the theta band and Fp2 and P3 in the beta band, as can be seen in Figure 12 and Figure 13, respectively (see positions framed by rectangles with dashed line). In this case, no separability was found in the alpha band.

The EEG signals and the bands found as discriminators between the state of relaxation (R) and the state determined by the arithmetic task (T) for each group of subjects are specified in Table 11 and Table 12.

To evaluate the performance of our analysis we used a set of established measures suitable for statistical models [7,9]:

(4) $$Accuracy=\frac{TP+TN}{TP+FP+TN+FN}$$

(5) $$Precision=\frac{TP}{TP+FP}$$

(6) $$MCC=\frac{\left(TP\times TN\right)-(FP\times FN)}{\sqrt{\left(TP+FP)(TP+FN)(TN+FP)(TN+FN\right)}}$$

(7) $$F1-score=\frac{2\times TP}{2\times TP+FP+FN}$$

(8) $$Sensitivity=\frac{TP}{TP+FN}$$

(9) $$Specificity=\frac{TN}{TN+FN}$$

These are defined in terms of the status of the spectral variable values in relation to the domains defining the mental state classes. The status of the variable is defined as follows:

- False positive (FP): *IF any value for test T ≤ MAX (all values for test R);*
- False negative (FN): *IF any value for test R ≥ MIN (all values for test T);*
- True positive (TP): *Number of Subjects—FN;*
- True negative (TN): *Number of Subjects—FP.*

Matthew’s correlation coefficient (MCC) is an aggregate measure to evaluate the precision of a model built from data. It considers all four categories (TP, TN, FP, and FN) and provides a more balanced view of the model’s performance. Basically, MCC provides a value between +1 and −1, where +1 means the best agreement between the predicted and current values and 0 means no agreement.

## 5. Results, Discussion, and Interpretations

A synthesis of the results obtained from the two types of analyses performed with the aim of finding EEG signals capable of discriminating mental state T in relation to R is presented in Table 13.

The EEG signals identified as discriminating in the current study are revealed and mentioned in several recent works that deal with this issue. These approaches, which also utilized data from PhysioNet, are summarized in Table 14.

First of all, we find from the reports that through different classification methods, using various signal analysis characteristics, a particular group of signals was identified as the most relevant for the detection and characterization of the mental arithmetic task. This group of signals is captured from several areas of the head (prefrontal, frontal, central, parietal, temporal, occipital). This fact is in agreement with the mapping of brain areas presented in Section 3.2, with reference to the neurodynamics of the brain.

Secondly, we will discuss the signals identified in our study as being the most sensitive to neural processes determined by mental arithmetic tasks. Thus, the most relevant discriminating signal, in accordance with the spectral peak criterion, for the G* group appears to be **F7** in both the theta and beta bands, and also the **Fz** signal in the theta band. So, these signals with 100% performance appear to classify the first 11 subjects that accomplished the task with high scores. For the whole of group G (25 subjects), signal F7 appears with 84% accuracy in theta, and Fz provides 90% accuracy. To improve discrimination, the correlation criterion for the F7–F4 pair can be considered. 

The **F3** signal in the theta band is also a good discriminator for group G* with 92% accuracy, and also for group G with 86% accuracy. The F3 signal appears with 100% accuracy for group B, and we are going to provide a possible explanation for that further on.

Also, to improve discrimination, the correlation criterion for the F8–F3 pair can be considered in this case. It was noticed that the F8 signal is 100% relevant as a group B discriminator for the spectral criterion in the theta band, as well as for the correlation criterion paired with Fp1 in the beta band. In addition, the **Fp1** signal appears as a discriminator according to the criterion of the spectral peak in the theta band, for group G, with an accuracy of 86%, while for group G*, it is a bit better, i.e., 94%.

An interesting status is found for the **Fp2** signal only for the spectral peak criterion, being discriminating exclusively in the theta and beta bands for the G* group, with an accuracy of 94%, and exclusively in the beta band only for the B group with an accuracy of 100%.

The **P3** signal in the theta and beta bands appears as a discriminator according to the spectral peak criterion with highest accuracy only for group B. Additionally, paired with **C4**, in the beta band, it could improve discrimination for group B. Also, for group G* in the theta band, it can be considered with an accuracy of 84%.

Two relevant observations stand out: (i) the spectral peak criterion is remarkable only in the theta and beta bands (most frequently in theta); (ii) the criterion of correlation between certain signals is manifested only in the beta band.

The spectral peak criterion is good enough to detect mental tasks with high accuracy, especially for subjects strongly involved in task solving, like those belonging to the G* group. From the perspective, the 100% accuracy of group B can be explained in terms of the higher mental effort spent by these subjects for task solving, which in fact denotes a strong involvement.

All in all, the interpretations of these results can be discussed in the context of current knowledge about brain activity in general and about the neuronal processes that are activated during mental arithmetic tasks.

Fp2 is located in the prefrontal area, defined as the area of decision-making processes, generally active in arithmetic processes, which can justify its involvement in groups G and B. Found in the literature as being involved in the detection of states of ambiguity [18], the Fp2 signal could measure the effect of the subjects’ effort to give a final answer for group B.

F3 is a signal that measures activity in the DLPFC area. This area was identified as being more active in the left cerebral hemisphere in the process of discovering the correct results when solving some equations [23]. Moreover, the F3 signal is also sensitive to the so-called risk situations [28] generated by the dilemma, which would be explained especially for the subjects of group B as an effect of hesitation in the elaboration of the answer.

Also, the F3 signal, in correlation with the F8 signal that captures the activity in the VLPFC area responsible for solving uncertainties and disambiguation [48], can be considered a discriminator for the G group.

The F7 signal symmetrically captures (from the left hemisphere, symmetrical in relation to F8) the activity in the VLPFC area, so it responds to disambiguation processes, which justifies its activation detected for the subjects of group G.

The reciprocal connection between the DLPFC and VLPFC areas (dilemma–disambiguation) can explain the correlation manifestation between F3–F8 and F7–F4 detected in the beta band.

The P3 signal is mentioned in the literature as being sensitive to the so-called “recovery of numerical facts” processes from memory [35,36]. We detected the presence of the spectral peak in theta and beta for P3 and a P3–C4 correlation, in beta, only for group B. Both signals are captured from areas responsible for episodic and semantic memory (P3), and working memory (C4), respectively, which could suggest that the subjects in group B showed a greater memory effort, similar to the results in [49].

## 6. Conclusions

The analysis tools used in this work are basic; they can be easily applied to any mental task experiment and do not require great computation effort. First, the spectral method applied by us assumed the filtering of EEG signals with band-pass filters to extract the signals in the theta, alpha, and beta sub-bands, followed by the evaluation of the spectral peaks and the determination of their variation ranges for groups of subjects in a state of relaxation and during a mental task. The classification method proposed for separating mental states uses the comparison of the limits of the detected variation ranges. This is a transient classification method that has a higher rejection rate if it is applied only for a single criterion—in our case, the spectral peak. In this study, the application of the method confirmed the validity of some discriminating signals, also confirmed by other researchers. The accuracy of the method of comparing the limits of ranges is under the control of the analyst, in the sense that they can establish an acceptable degree of overlap of the respective limits, thus determining the accepted the level degree of confidence.

Second, the Pearson statistical correlation coefficient was used to detect some pairs of signals that correlate with an accepted threshold of the correlation coefficient, set in our case at 0.5 (accepted as average). This discrimination method does not offer a significant degree of confidence, but, interpreted in conjunction with the spectral peak criterion, it can provide pertinent explanations regarding mental activity in accordance with evidence recently reported in the field of neuroscience.

A series of considerations regarding the electrical activity of the brain were also confirmed in our study, such as the fact that the size of the spectral peak increases with the presence of mental load [8,10,50]. Its range of variation differs from subject to subject, depending somewhat on the intensity of the mental effort. Another confirmation in the present study is the fact that the statistical correlation between signals decreases in the presence of mental load as an effect of the well-known phenomenon of functional connectivity in the brain [13,17].

Also, this study highlights the relevance of theta and beta brain waves for the presence of arithmetic mental tasks. It is generally recognized that theta waves appear in the context of cognitive functions, being associated with the state of consciousness [33].

Finally, the current study makes a contribution by trying to explain the behavior of some EEG signals in accordance with the information about the neurodynamics of the brain in the functional areas responsible for manipulating numbers and performing arithmetic operations.

Meanwhile, we are guided by the results of this work and plan to conduct our own experiments for acquiring relevant EEG signals in discriminating mental arithmetic tasks and validating the proposed methods based on BCI concepts [5,51], into a practical approach on possible hardware solutions [52,53]. To further explore this phenomenon, we plan to extend the EEG signal recording capacity in future research by using a 64-channel EEG system from Biosemi, under experimental design conditions similar to those in the PhysioNet database. By analyzing the data obtained from a larger number of electrodes, we can identify potential differences or similarities to complement previous results and gain a more comprehensive understanding of the phenomenon under study.

## Figures and Tables

**Table 1:** Databases available for arithmetic task.

Name	Description
PhysioNet [18] https://physionet.org (accessed on 5 April 2023)	free access, arithmetic tasks consisting of subtraction operations, 19 channels
Keirn and Aunon [19]	free access, 6 EEG channels, 4 types of task: arithmetic—consisting of multiplication and imaginary visual counting—plus tasks associated with writing a letter and rotating geometric figures

**Table 2:** Summary of the experiment.

Item	Materials	Quantity	Details
Equipment	Neurocom (Kharkov, Ukraine, XAI-MEDICA) (Ag/AgCl electrodes)	23 channels (19 EEG + 2 reference A1-A2, 1ECG)	Monopolar Prefrontal (Fp1, Fp2), Frontal (F3, F4, Fz, F7, F8) Central (C3, C4, Cz), Parietal (P3, P4, Pz) Temporal (T3, T4, T5, T6), and Occipital (O1, O2) Sampling rate: 500 Hz Unique band-pass filter: 0.5–30 Hz
Subjects	Students (male and female) in the field of biology and medicine Average age = 18.6 years, standard deviation (SD) = 0.87 years)	36	26 subjects—Group “G”, the average number of operations performed per minute = 21 ± 7.4 10 subjects—Group “B”, the average number of operations performed per minute = 7 ± 3.6
Tests	Verbally communicate with the group of subjects	2	1 test—without the arithmetic task, in relaxed state, with closed eyes (180 s) 1 test—with arithmetic tasks (60 s)

**Table 3:** The groups of subjects in order of performance of the answers, with the top 11 subjects (shaded) scoring above the group average.

Group	No.	Subject ID	Number of Subtractions	Gender F–Female M—Male	Age
G	1.	Subject 27	34.59	F	19
	2.	Subject 13	34	M	24
	3.	Subject 3	31	F	17
	4.	Subject 34	31	F	18
	5.	Subject 25	30.53	M	17
	6	Subject 1	29.35	F	18
	7.	Subject 17	28.7	F	17
	8.	Subject 23	27.47	F	16
	9.	Subject 28	27	F	19
	10.	Subject 12	26.36	F	17
	11.	Subject 11	26	F	18
	12.	Subject 15	22.18	F	17
	13.	Subject 33	21.47	M	17
	14.	Subject 5	20.71	F	16
	15.	Subject 18	20	F	17
	16.	Subject 31 (Excluded)	19.88	F	19
	17.	Subject 8	18.24	M	26
	18.	Subject 29	16.59	M	19
	19.	Subject 20	15.41	F	17
	20.	Subject 24	14.76	M	17
	21.	Subject 26	13.59	F	17
	22.	Subject 7	13.38	F	18
	23.	Subject 32	13	F	20
	24.	Subject 2	12.88	F	19
	25.	Subject 35	12.18	F	17
	26.	Subject 16	11.59	F	17
B	1.	Subject 30	10	F	17
	2.	Subject 0	9.7	M	21
	3.	Subject 14	9	F	17
	4.	Subject 4 (Excluded)	8.6	F	18
	5.	Subject 19	7.06	M	17
	6.	Subject 9	7	F	18
	7.	Subject 22	4.47	F	17
	8.	Subject 6	4.35	F	16
	9.	Subject 10	1	F	19
	10.	Subject 21	1	F	17

**Table 4:** Number of signal combinations fulfilling the condition related to correlation threshold.

Signal Type		Experimental Group G (25)					Experimental Group B (9)				
		c_corr R > 0.5	c_corr R > 0.6	c_corr R > 0.7	c_corr R > 0.8	c_corr T < 0.5	c_corr R > 0.5	c_corr R > 0.6	c_corr R > 0.7	c_corr R > 0.8	c_corr T < 0.5
Raw		32	15	1	0	23	33	17	7	1	23
Filtered	theta	33	15	3	0	1	33	24	6	1	23
	alpha	30	15	5	2	26	39	27	14	2	26
	beta	25	10	3	0	40	31	13	3	0	52

**Table 5:** Number of subjects (from group G) fulfilling Equation (3).

Band	Paired Signals	Fulfillment Conditions	
		c_corr R > 0.5	c_corr T < 0.5
β	F7–F4	16	15
	F8–F3	15	16
	T3–Fp1	17	15
	Pz–T4	14	13

**Table 6:** Subjects (from group G) identified in each group in the beta band.

Paired Signals	Subject with c_corr R > 0.5 Set N	Subjects with c_corr T < 0.5 Set M	Common Subjects (CSs)
F7–F4	16 subjects = {S1, S3, S7, **S15**, **S16**, S17, **S20**, **S23**, S24, **S26**, S27, **S28**, S29, S32, S33, S34}	15 subjects = {S2, S5, S8, S11, S12, S13, **S15**, **S16**, S18, **S20**, **S23**, S25, **S26**, **S28**, S35}	{S15, S16, S20, S23, S26, S28}
F8–F3	15 subjects = {S1, **S3**, S7, **S11**, **S15**, S17, **S20**, S23, S24, S26, **S27**, **S28**, S29, **S32**, S33}	16 subjects = {S2, **S3**, S8, **S11**, S12, S13, **S15**, S16, S18, **S20**, S25, **S27**, **S28**, **S32**, S34, S35}	{S3, S11, S15, S20, S27, S28, S32}

**Table 7:** Hierarchy according to the subjects’ performance.

F7–F4		F8–F3	
Subject	Score	Subject	Score
**S23**	27.47	**S27**	34.59
**S28**	27	**S3**	31
**S15**	22.18	**S28**	27
S20	15.41	**S11**	26
S26	13.59	**S15**	22.18
S16	11.59	S20	15.41
		S32	13

**Table 8:** Number of subjects (from group B) who fulfill Equation (3).

Band	Paired Signals	Fulfillment Conditions	
		c_corr R > 0.5	c_corr T < 0.5
β	F7–F4	5	6
	F8–Fp1	5	6
	P3–C4	6	5
	Pz–T5	5	6

**Table 9:** Subjects (from group B) identified in each group in beta band.

Pair Signals	Subject with c_corr R > 0.5 Set N	Subjects with c_corr T < 0.5 Set M	Common Subjects (CSs)
F7–F4	5 subjects = {S0, **S19**, S21, S22, **S30**}	6 subjects = {S6, S9, S10, S14, **S19**, **S30**}	{S19, S30}
F8–Fp1	5 subjects = {**S0**, S6, **S19**, S21, **S30**}	6 subjects = {**S0**, S9, S10, S14, **S19**, **S30**}	{S0, S19, S30}
P3–C4	6 subjects = {S0, S6, **S9**, **S19**, S21, **S22**}	5 subjects = {**S9**, S10, S14, **S19**, **S22**}	{S9, S19, S22}
Pz–T5	5 subjects = {S0, S6, S9, **S21**, S22}	6 subjects = {S9, S14, S19, **S21**, S30}	{S21}

**Table 10:** Hierarchy (for group B) by performance of common subjects.

F7–F4		F8–Fp1		P3–C4		Pz–T5	
Subject	Score	Subject	Score	Subiect	Score	Subiect	Score
S30	10	S30	10	S9	7	S21	1
S19	7.06	S0	9.7	S19	7.06		
		S19	7.06	S22	4.47		

**Table 11:** Discriminators for resting state vs. mental task for the subjects with scores ≥ 26 (group G*).

Band	Signal
θ	F7
	Fz
α	-
β	F7

**Table 12:** Discriminators for resting state vs. mental task for the subjects of group “Bad”.

Band	Signal
θ	F3
	F8
	P3
α	-
β	Fp2
	P3

**Table 13:** Synthesis with potentially discriminating signals.

EEG Signal	Criterion: Spectral Peak							
	Performance							
	Band	Group	Accuracy (%)	Precision (%)	MCC	F1 Score (%)	Sensitivity (%)	Specificity (%)
F7	θ	G*	100	100	1	100	100	100
		G	84	79	0.69	85	92	76
		B	77.78	73	0.57	80	89	67
	β	G*	100	100	1	100	100	100
		G	62	59	0.26	68	80	44
		B	61.11	57	0.27	70	89	33
Fz	θ	G*	100	100	1	100	100	100
		G	90	92	0.80	90	88	92
		B	88.89	89	0.78	89	89	89
F3	θ	G*	92	96	0.84	92	88	96
		G	86	88	0.72	86	84	88
		B	100	100	1	100	100	100
F8	θ	G*	82	75	0.67	84	96	68
		G	56	53	0.17	68	92	20
		B	100	100	1	100	100	100
Fp1	θ	G*	94	96	0.88	94	92	96
		G	86	91	0.73	85	80	92
		B	83.33	80	0.67	84	89	78
Fp2	θ	G*	94	96	0.88	94	92	96
		G	70	78	0.42	65	56	84
		B	83.33	88	0.67	82	78	89
	β	G*	94	96	0.88	94	92	96
		G	62	59	0.25	67	76	48
		B	100	100	1	100	100	100
P3	θ	G*	84	84	0.68	84	84	84
		G	48	48	−0.04	55	64	32
		B	100	100	1	100	100	100
	β	G*	72	76	0.68	70	64	80
		G	20	14	−0.61	13	12	28
		B	100	100	1	100	100	100

**Table 14:** A synthesis of reported results on signal classification based on the PhysioNet database.

Classification Method	Features Used	Accuracy	Signals	Reference
DT	Energies, entropies, averages, L2 norm in sub-bands	>95% for R vs. T	beta: F7, Cz alpha: Cz, O1	[2]
SVM	Fourier analysis results, energy, entropy, variance	98.6–90.3% for R vs. T	C3, F4, Fp2, P3, O1, T3	[3]
KNN	Hybrid characteristics between frequency distribution and root mean square amplitude and coefficients of a sixth-order autoregressive model	99.74–97.82% for R vs. T 99.93–99.94%	Fp1, Fp2, F3, F7, Fz, F8, F4,	[4]
LDA			Fp1, Fp2	
RNN	Time–frequency characteristics, spectral power in sub-bands (δ, θ, α, β, γ)	93.59–91.67% LSTM for R vs. T	Fp1, F7, Pz, P4	[5]
RNN	Entropies	99.63–99.38% LSTM, BLSTM, GRU for R vs. T	O2, Fz, Cz, Pz	[6]
A comparative method	Statistical features from PSD, DFA and coherencein sub-bands (θ, β)	Unspecified for R vs. T	F7, T3, T5 O1, T5, P3, T6	[8]
The domain limits of sprectral peak and the correlation threshold	The peak of the spectral power in θ, α, β bands, the Pearson statistical correlation coefficient	84–100% for R vs. T max. 30%	F7, Fz, F3,F8, Fp1, Fp2 F4, T3, and C4; they also appear as paired signals in the correlation criterion	This paper

## Data Availability

The codes of this paper are available by request to the authors.
